# The Predictive Validity of the Danish Psychosocial Work Environment Questionnaire With Regard to Onset of Depressive Disorders and Long-Term Sickness Absence

**DOI:** 10.1093/annweh/wxac069

**Published:** 2022-10-15

**Authors:** Thomas Clausen, Karl Bang Christensen, Jeppe Karl Sørensen, Jakob B Bjorner, Ida E H Madsen, Vilhelm Borg, Reiner Rugulies

**Affiliations:** National Research Centre for the Working Environment, Copenhagen, Denmark; Department of Public Health, University of Copenhagen, Copenhagen, Denmark; National Research Centre for the Working Environment, Copenhagen, Denmark; National Research Centre for the Working Environment, Copenhagen, Denmark; Department of Public Health, University of Copenhagen, Copenhagen, Denmark; Optum Patient Insights, Johnston, RI, USA; National Research Centre for the Working Environment, Copenhagen, Denmark; National Research Centre for the Working Environment, Copenhagen, Denmark; National Research Centre for the Working Environment, Copenhagen, Denmark; Department of Public Health, University of Copenhagen, Copenhagen, Denmark

**Keywords:** job characteristics, occupational health, psychosocial work environment, questionnaire, stress, survey, validity, work characteristics, working conditions

## Abstract

**Objectives:**

To investigate the predictive validity of 32 measures of the Danish Psychosocial Work Environment Questionnaire (DPQ) against two criteria variables: onset of depressive disorders and long-term sickness absence (LTSA).

**Methods:**

The DPQ was sent to 8958 employed individuals in 14 job groups of which 4340 responded (response rate: 48.4%). Depressive disorders were measured by self-report with a 6-month follow-up. LTSA was measured with a 1-year follow-up in a national register. We analyzed onset of depressive disorders at follow-up using logistic regression models, adjusted for age, sex, and job group, while excluding respondents with depressive disorders at baseline. We analyzed onset of LTSA with Cox regression models, adjusted for age, sex, and job group, while excluding respondents with previous LTSA.

**Results:**

The general pattern of the results followed our hypotheses as high job demands, poorly organized working conditions, poor relations to colleagues and superiors, and negative reactions to the work situation predicted onset of depressive disorders at follow-up and onset of LTSA during follow-up. Analyzing onset of depressive disorders and onset of LTSA, we found risk estimates that deviated from unity in most of the investigated associations. Overall, we found higher risk estimates when analyzing onset of depressive disorders compared with onset of LTSA.

**Conclusions:**

The analyses provide support for the predictive validity of most DPQ-measures. Results suggest that the DPQ constitutes a useful tool for identifying risk factors for depression and LTSA in the psychosocial work environment.

**What’s important about this paper**

Questionnaires are important tools for assessing psychosocial working conditions and their validity and reliability should be rigorously tested. Building on prior work that explored the construct validity and reliability of the Danish Psychosocial Work Environment Questionnaire (DPQ), this study assessed the predictive validity of the DPQ against two criteria variables: a self-reported measure of depressive disorders and a register-based measure of long-term sickness absence. Overall, this study found satisfactory predictive validity of the measures of the DPQ, as the general pattern of the results complied with our hypotheses. The DPQ can be used to identify risk factors for adverse health outcomes in the psychosocial work environment.

## Introduction

When analyzing the psychometric properties of scales and single items in questionnaires, it is important to investigate the validity and the reliability of the measures (Cronbach and Meehl, 1955; Mokkink *et al.*, 2010). In evaluating the validity of questionnaire-based measures it is also important to investigate whether the measures under study are associated with relevant outcomes in ways that comply with hypotheses derived from prevalent theoretical perspectives and existing empirical evidence (Cronbach and Meehl, 1955; Messick, 1995; Fayers and Machin, 2000). When doing prospective analyses, this is referred to as *predictive validity* (Cronbach and Meehl, 1955). In this study, we investigate the predictive validity of a series of measures that have been developed for the Danish Psychosocial Work Environment Questionnaire (DPQ) (Clausen *et al.*, 2019).

The DPQ operationalizes 38 dimensions of the psychosocial work environment and extant research points to the relevance of the development of a broad range of validated questionnaire-based measures to assess psychosocial working conditions in contemporary workplaces (Rugulies *et al.*, 2010; Clausen *et al.*, 2014a; Theorell *et al.*, 2015; Finne *et al.*, 2016; Duchaine *et al.*, 2020; Niedhammer *et al.*, 2021). Indeed, numerous studies have shown associations between different factors in the psychosocial work environment and various outcomes that are related to the health and well-being of employees. A systematic review of longitudinal studies (Theorell *et al.*, 2015) suggests that adverse psychosocial working conditions, such as low influence at work (decision latitude), exposure to workplace bullying, low social support, limited skill discretion, high psychological demands, job insecurity, and low levels of organizational justice are associated with an increased risk of depression. Other studies indicate that low levels of influence at work, poor quality of leadership, emotional demands, role conflicts, and experience of low levels of meaning at work are prospectively associated with increased risk of long-term sickness absence (LTSA) (Rugulies *et al.*, 2010; Clausen *et al.*, 2014a,b). This cursory review of the literature suggests that a variety of factors in the psychosocial work environment are important determinants not only of more or less immediate affective states related to the psychological well-being of employees but also more tangible labor market outcomes, such as risk of sickness absence.

To facilitate continued high-quality research in psychosocial working conditions, the National Research Centre for the Working Environment (NRCWE) has updated its’ questionnaire on psychosocial working conditions. This decision was based on a desire to update the questionnaire that has hitherto formed the basis of NRCWE’s research in psychosocial working conditions [Copenhagen Psychosocial Questionnaire (COPSOQ II); Pejtersen *et al.*, 2010] in order to (i) capture and operationalize emerging issues within the field of psychosocial work environment, and (ii) to enhance the methodological quality of the measures applied in psychosocial work environment research.

We previously reported that the construct validity and the internal consistency and test–retest reliability of the DPQ was satisfactory (Clausen *et al.*, 2019). Our assessment of the construct validity was based on cross-sectional comparisons of the DPQ-measures between job groups, on analyses of factorial validity, and on analyses of discriminant validity.

The general finding of the previous analyses was that the measures of the DPQ were valid and reliable *within* each of the 14 job groups in the validation study, which implies that the measures are applicable in a wide range of different job groups.

In the present study, we assess the predictive validity of the DPQ-measures. An assessment of the predictive validity contributes to the overall assessment of the validity of measures by ascertaining prospective associations between the measures that are being tested and relevant criteria variables. According to Messick (1995), when investigating the predictive validity of questionnaire-based measures, the observed associations between predictor variables and criteria variables ‘should make theoretical sense in terms of what the predictor test is interpreted to measure and what the criterion is supposed to embody.’ (p. 744). In this study, we deploy two criteria variables; one self-reported measure of depressive disorders (Bech *et al.*, 2001) and a register-based measure of LTSA (Hjollund *et al.*, 2007). We hypothesize that all measures of the DPQ are associated with the two criteria variables, and Table 1 gives an overview of the hypothesized directions of the associations for each of the measures in the DPQ.

**Table 1.** Hypothesized associations between higher levels of the measures in the DPQ and the two criteria variables.

Name of scale/single item	Expected direction of association with risk of depressive disorder	Main reference	Expected direction of association with risk of LTSA	Main reference
Domain: Demands at work				
Quantitative demands	Higher risk	Theorell *et al.* (2015)	Lower risk	Clausen *et al.* (2014a)
Work pace	Higher risk	Theorell *et al.* (2015)	Higher risk	Clausen *et al.* (2014a)
Emotional demands	Higher risk	Vammen *et al.* (2016)	Higher risk	Framke *et al.* (2019)
Demands to conceal feelings	Higher risk	Zapf (2002)	Higher risk	Rugulies *et al.* (2010)
Cognitive demands	Higher risk	No ref.	Higher risk	Rugulies *et al.* (2010)
Work without boundaries	Higher risk	Albertsen and Garde (2009)	Higher risk	Rugulies *et al.* (2010)
Domain: Work organization and job content				
Influence at work	Lower risk	Theorell *et al.* (2015)	Lower risk	Clausen *et al.* (2014a)
Influence on working hours	Lower risk	Ala Mursula *et al.* (2004)	Lower risk	No refs.
Possibilities for development	Lower risk	Theorell *et al.* (2015)	Lower risk	Rugulies *et al.* (2010)
Role clarity	Lower risk	Clausen and Borg (2011)	Lower risk	No ref.
Role conflicts	Higher risk	Finne *et al.* (2016)	Higher risk	Rugulies *et al.* (2010)
Predictability	Lower risk	Finne *et al.* (2016)	Lower risk	No ref.
Possibilities for performing work tasks	Lower risk	Sasser and Sørensen (2016)	Lower risk	No ref.
Unnecessary work tasks	Higher risk	Madsen *et al.* (2014)	Higher risk	No ref.
Domain: Interpersonal relations: cooperation and leadership				
Social support from colleagues	Lower risk	Theorell *et al.* (2015)	Lower risk	Roelen *et al.* (2018)
Cooperation between colleagues within teams, departments, or groups	Lower risk	Theorell *et al.* (2015)	Lower risk	Clausen *et al.* (2020)
Trust between colleagues	Lower risk	Guinot *et al.* (2014)	Lower risk	Clausen *et al.* (2020)
Social support from management	Lower risk	Finne *et al.* (2016)	Lower risk	Sørensen et al. (2020)
Quality of leadership	Lower risk	Nielsen and Daniels (2012)	Lower risk	Sørensen et al. (2020)
Cooperation with immediate supervisor	Lower risk	Nielsen and Daniels (2012)	Lower risk	Clausen *et al.* (2020)
Justice in the workplace	Lower risk	Elovainio *et al.* (2015)	Lower risk	Leineweber *et al.* (2017)
Involvement of employees	Lower risk	No ref.	Lower risk	No ref.
Changes in the workplace	Lower risk	Bambra *et al.* (2007)	Lower risk	No ref.
Recognition	Lower risk	Nieuwenhuijsen *et al.* (2010)	Lower risk	Rugulies *et al.* (2010)
Domain: Reactions to the work situation				
Experience of meaning at work	Lower risk	Burr *et al.* (2010)	Lower risk	Clausen *et al.* (2014b)
Commitment to the workplace	Lower risk	Finne *et al.* (2016)	Lower risk	Clausen *et al.* (2014b)
Work engagement	Lower risk	Halbesleben (2010)	Lower risk	Rongen *et al.* (2014)
Job insecurity	Higher risk	Theorell *et al.* (2015)	Higher risk	Antai *et al.* (2015)
Self-reported stress	Higher risk	LaMontagne *et al.* (2010)	Higher risk	Pedersen *et al.* (2021)
Job satisfaction	Lower risk	Bowling *et al.* (2010)	Lower risk	Roelen *et al.* (2008)
Overall assessment of the psychosocial work environment	Lower risk	No ref.	Lower risk	No ref.
Conflict between work life and private life	Higher risk	Amstad *et al.* (2011)	Higher risk	Antai *et al.* (2015)

The DPQ-measures 38 dimensions of the psychosocial work environment that are subdivided into five overall domains: (i) Demands at work, (ii) Work organization and job content, (iii) Interpersonal relations: cooperation and leadership, (iv) Conflicts and negative acts in the workplace, and (v) Reactions to the work situation. In this study, we focus on the 32 dimensions of the DPQ that are measured as continuous variables and we thereby exclude the six measures from the domain ‘Conflicts and negative acts in the workplace’. We exclude these measures from the present analysis for two reasons: First, four of the six measures in this domain are not new and the predictive validity of these measures has, therefore, been tested previously (Clausen *et al.*, 2012; Rugulies *et al.*, 2020). Second, the six measures are measured as dichotomous variables, while the remaining 32 DPQ-measures are measured as continuous variables and, accordingly, the results from the analyses of the dichotomous and continuous variables are not directly comparable.

Moreover, it is likely that the dimensions the DPQ are interrelated and that some dimensions may constitute mediators or moderators in the association between other dimensions of the DPQ and the two outcome variables. However, as *the aim of this study is to assess the predictive validity of the DPQ-measures*, this study will have its’ focus on examining prospective associations between each of the 32 DPQ-measures and the two outcome variables (onset of depressive disorders and LTSA), while adjusting for potential confounders.

We deploy three conditions in our assessment of predictive validity: (i) does the association between the individual measures of the DPQ and the two criteria variables point in the directions, hypothesized in Table 1?, (ii) is the association practically relevant?, and (iii) is the association statistically significant?

## Methods

This study is based on data from a prospective cohort study on employed individuals in Denmark. The baseline questionnaire was distributed from April to June in 2015 and after 6 months, a follow-up questionnaire was sent to the respondents of the baseline questionnaire. We further merged data from the baseline study with the Danish Register for Evaluation of Marginalisation (DREAM) (Hjollund *et al.*, 2007) that includes weekly updated information of all social transfer payments, including sickness absence benefits.

### Study population and procedures

The baseline questionnaire was distributed to a population of 8958 employed individuals. The study population was stratified by educational attainment (no/low-level education, medium-level education, and high-level education) and primary work task (work related to the processing of knowledge, client-related work, work related to production and transportation, and work related to sales and marketing) of the respondents. When selecting the population for the baseline study, we included two job groups representing ‘work related to production and transportation’ with no or low levels of educational attainment: ‘slaughterhouse workers’ and ‘mail carriers’. We also included the job group ‘police officers’ to represent client-related work tasks, as this job group has the direct possibility to apply force toward the clients they deal with, which distinguishes this job group from the other job groups in this category (healthcare helpers, school teachers, and medical doctors). Accordingly, the study population was based on 14 job groups that represent a variety of positions in the Danish labor market. Procedures for sampling and questionnaire interviews are described in detail elsewhere (Clausen *et al.*, 2019).

Of the 8958 invited individuals, 4340 responded (48.4%). Response rates varied between 35.3% (slaughterhouse workers) and 61.6% (technical draughtsmen) across the 14 job groups (Table 2). Participants were informed that the purpose of the survey was to develop a new questionnaire containing questions about the respondents’ work, psychosocial work environment, and well-being.

**Table 2.** Description of the study population in the 14 job groups.

Job group	Baseline study					Follow-up study	
	Invited	Participants	Response rate (%)	Age [Mean (SD)]	Female sex (%)	Participants	Response rate (%)a
1. Office workers	592	308	52.0	46.5 (11.7)	83.4	185	60.1
2. Technical draughtsmen	536	330	61.6	48.6 (10.2)	56.7	203	61.5
3. Teaching and research staff in universities	590	294	49.8	43.2 (13.4)	47.3	172	58.5
4. Healthcare helpers	563	248	44.0	49.8 (12.5)	94.0	131	52.8
5. Primary school teachers	559	321	57.4	46.5 (11.0)	71.0	212	66.0
6. Medical doctors	490	267	54.5	45.3 (11.8)	47.6	174	65.2
7. Mail carriers	560	287	51.3	47.0 (12.1)	34.5	158	55.1
8. Slaughterhouse workers	935	330	35.3	48.3 (9.6)	16.1	171	51.8
9. Smith workers	647	260	40.2	46.9 (12.0)	1.5	143	55.0
10. Engineers (construction)	611	350	57.3	46.3 (12.1)	20.6	231	66.0
11. Sales assistants in shops	904	323	35.7	34.0 (14.2)	63.2	151	46.7
12. Private bankers	766	378	49.3	44.6 (12.4)	77.8	206	54.5
13. Business managers	600	332	55.3	50.3 (8.0)	39.8	203	61.1
14. Police officers	605	312	51.6	47.5 (10.7)	14.7	200	64.1
Total	8958	4340	48.4	46.0 (12.2)	47.8	2540	58.5

Response rate in the follow-up study was calculated as the number of participants at follow-up divided by number of participants at baseline.

An analysis of non-response at baseline showed that women were significantly more likely to participate than men in 6 of the 14 job groups and that older respondents were significantly more likely to participate than younger respondents in all of the 14 job groups (Clausen *et al.*, 2019).

Approximately 6 months after the baseline study [mean follow-up time was 165 days (SD = 37)], we sent a follow-up questionnaire to the 4319 eligible participants from the baseline study. Twenty-one participants from the baseline study were ineligible for participation in the follow-up study due to retirement, address protection, emigration, or death. The follow-up study followed the same procedures as the baseline study and we obtained response from 2540 employees (58.5%). Across the 14 job groups, response rates varied between 52.8% (healthcare helpers) and 66.0% (engineers in construction) (Table 2). An analysis of attrition from loss of respondents from baseline to follow-up showed no statistically significant differences between men and women in any of the 14 job groups. In 9 of the 14 job groups, older respondents were significantly more likely to respond to the follow-up survey than younger respondents (results not shown). Further analyses of attrition from baseline to follow-up showed no statistically significant differences in prevalence of LTSA during follow-up or in the prevalence of depressive disorders at baseline for participants and non-participants in the follow-up study (results not shown).

### Measures

We included 32 DPQ-measures in this study. Items and internal consistency reliabilities of the multi-items scales of the DPQ are described in Appendix 1 (Supplementary Material, available at *Annals of Work Exposures and Health* online). The test–retest reliability and construct validity (in particular factorial validity and discriminant validity) of the measures of the DPQ were also assessed using the *baseline* survey of the present study and have been reported elsewhere (Clausen *et al.*, 2019).

The measures of the DPQ were rescaled into scores ranging from 0 to 100, with higher values indicating higher levels of the measured dimension.

We measured onset of depressive disorders using the *Major Depression Inventory* (MDI) (Bech *et al.*, 2001). The MDI consists of 12 items assessing the presence of depressive symptoms during the last 2 weeks. A sample item is: ‘How much of the time during the past two weeks have you felt in low spirits or sad?’ Response options ranged from 0 (the symptom has not been present at all) to 5 (the symptom has been present all of the time). The MDI sum score ranges from 0 to 50 points, as for two pairs of items only the higher score is considered. A clinical validation study has previously shown that an MDI-score ≥21 indicates a depressive disorder (Bech *et al.*, 2015), and, accordingly, we used this MDI-score for defining the presence of depressive disorders in this study.

Data on LTSA were collected from the DREAM-register (Hjollund *et al.*, 2007) and was defined as 6 or more consecutive weeks of sickness absence in the 12-month follow-up period after baseline. Data from the baseline survey were linked to the DREAM-register using the respondents’ unique civil registration number and the linkage was successful for all 4340 respondents. Information on age, sex, and job groups was retrieved from a national income register (e-Indkomstregistret).

### Statistical analyses

We pooled all respondents in the analyses, due to the low number of respondents within each job group.

We analyzed prospective associations between the DPQ-measures at baseline and depressive disorders at follow-up using logistic regression models to estimate odds ratios and 95% confidence intervals (95% CIs). All analyses were adjusted for age, sex, and job group and we excluded respondents with a depressive disorder (MDI-score ≥21) at baseline. Associations were analyzed using the LOGISTIC procedure in SAS 9.4 (SAS Institute, Cary, NC).

We analyzed associations between the measures of the DPQ and LTSA using Cox regression models to estimate hazard ratios and 95% CIs. We defined LTSA during a 12-month follow-up period with calendar time measured in weeks as the underlying time axis. Participants were followed from the week where they answered the questionnaire until first onset of LTSA or censoring due to migration, retirement, death, or end of follow-up. We excluded respondents with LTSA for a 24-month period prior to baseline. All analyses were adjusted for age, sex, and job group. We found that the proportional hazard assumption was satisfied in all analyses through visual inspection of Schoenfeld Residual plots and negative log of the estimated survival curve across each of the 32 DPQ-measures divided into quartiles. Data were analyzed using the PHREG procedure in SAS 9.4 (SAS Institute, Cary, NC).

Sex and job group were entered into the analyses as nominal variables. The 32 DPQ-measures were entered into the models as continuous variables. All predictors were standardized.

Since a total of 32 tests were performed for each of the two outcomes, we interpreted *P* values using the Benjamini–Hochberg correction for multiple testing (Benjamini and Hochberg, 1995) with the MULTTEST procedure in SAS 9.4 (SAS Institute, Cary, NC).

## Results

### Outcome: onset of depressive disorders

**Figure 1.**
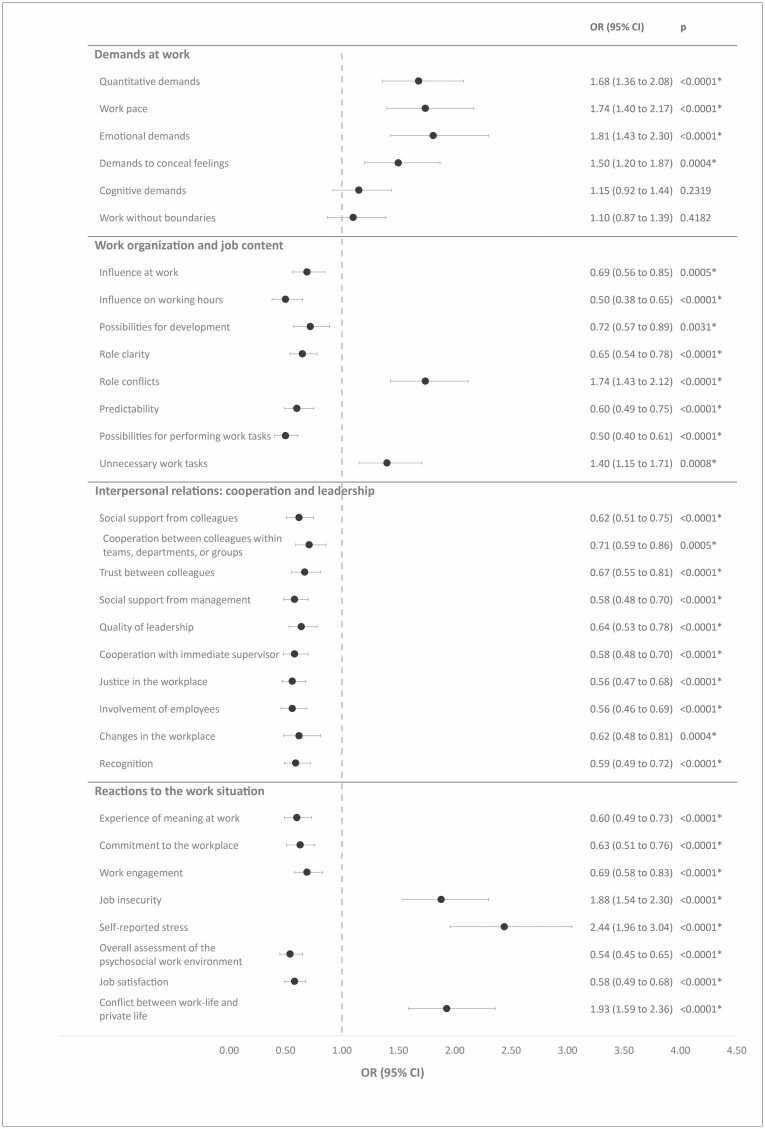
Prospective associations between measures of the DPQ and onset of depressive disorders. Note: OR, odds ratio. Associations were adjusted for age, sex, and job group. Participants with depressive disorders at baseline (MDI ≥21) were excluded from the analysis. *Statistically significant after adjusting for multiple testing.

Fig. 1 shows the results from the prospective analysis of associations between measures from the DPQ and onset of depressive disorders at follow-up, adjusted for age, sex, and job group. After excluding 188 respondents with depressive disorders at baseline (8.2%), 101 respondents reported a depressive disorder at follow-up (4.8%).

In the domain *Demands at work*, we found, in accordance with our hypotheses, that higher levels of the six multi-item scales at baseline predicted onset of depressive disorders at follow-up. We found that an increase of 1 SD of the measures *quantitative demands*, *work pace*, *emotional demands*, *demands to conceal feelings* predicted increased odds ratios varying between 1.50 and 1.81 with CIs clearly deviating from unity. For the latter two measures (*cognitive demands* and *work without boundaries*), we found elevated odds ratios that did not depart from unity.

In the domain *Work organization and job content*, all eight multi-items scales were prospectively associated with psychological well-being at follow-up. The results indicate that higher levels of *role conflicts* and *unnecessary work tasks* at baseline predicted onset of depressive disorders. For the other measures in this domain, we found that higher scale values at baseline predicted a decreased risk of depressive disorders at follow-up. All findings were in accordance with our hypotheses and all odds ratios departed from unity.

In the domain *Interpersonal relations: cooperation and leadership*, we found, in accordance with our hypotheses, that an increase of 1 SD in the nine multi-item scales and the one single-item predicted a decreased risk for onset of depressive disorders at follow-up. Odds ratios varied between 0.56 and 0.71 and the observed CIs deviated from unity.

All observed associations between the measures within the domain *Reactions to the work situation* and onset of depressive disorders were in accordance with our hypotheses. The results show that an increase of 1 SD in the multi-item scales *job insecurity*, *stress*, and *conflict between work and private life* predicted onset of depressive disorders, with odds ratios varying between 1.88 and 2.44 and the CIs clearly deviating from unity. For the remaining measures in this domain, we found that higher scale values predicted reduced risk of depressive disorders at follow-up. Odds ratios varied between 0.54 and 0.69 with CIs clearly deviating from unity.

Fig. 1 shows that 30 of the 32 associations we statistically significant. All statistically significant associations remained statistically significant after Benjamini–Hochberg correction for multiple testing.

### Outcome: onset of LTSA

**Figure 2.**
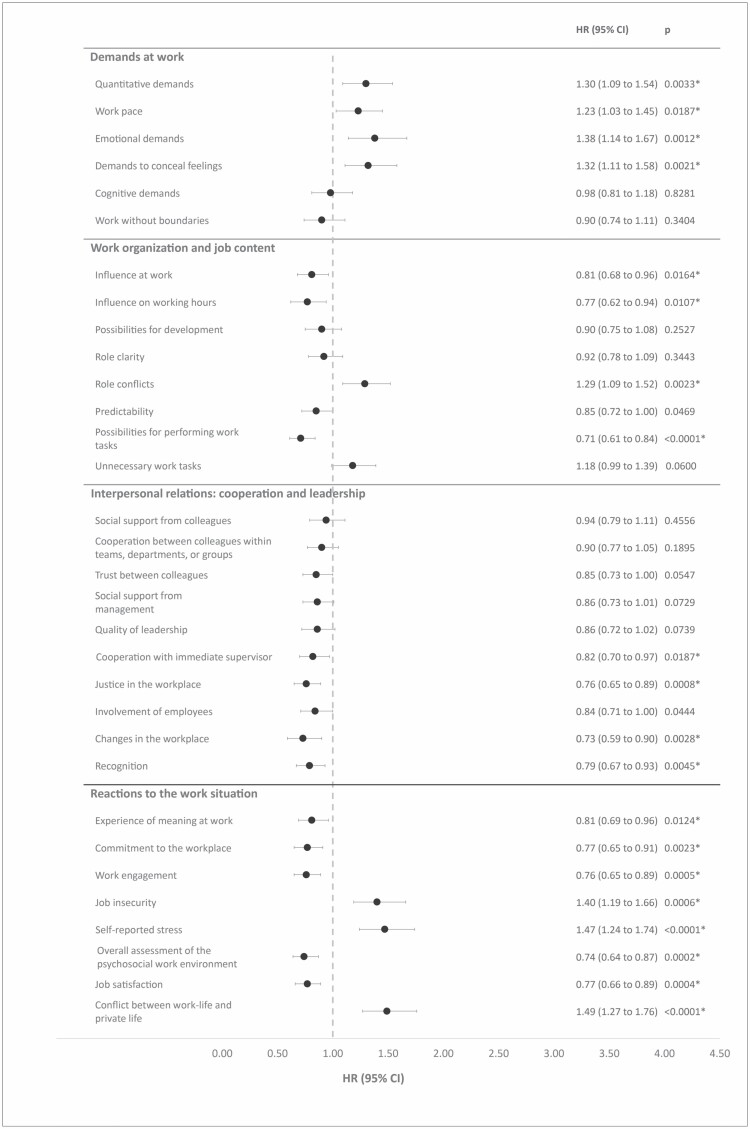
Prospective associations between measures of the DPQ and onset of LTSA. Note: HR, hazard ratio. Associations were adjusted for age, sex, and job group. Participants with LTSA for 24 months prior to baseline were excluded from the analysis. *Statistically significant after adjusting for multiple testing.

In Fig. 2, we present results from the prospective analysis of associations between measures from the DPQ and risk of LTSA. During 3973 person years, we identified 235 cases of LTSA (59 cases per 1000 person years).

Within the domain *Demands at work*, we found that an increase of 1 SD on the multi-item scales *quantitative demands*, *work pace*, *emotional demands*, and *demands to conceal feelings* were associated with hazard ratios for LTSA varying between 1.23 and 1.38. The CIs deviated from unity. For *quantitative demands*, the results countered the expected direction of the association, whereas the remaining results were in accordance with our hypotheses. For the remaining measures in this domain, hazard ratios were close to unity, but the results indicate that respondents with high scores on the scale *work without boundaries* had a reduced risk of LTSA although the CI did not depart from unity. This finding counters our hypothesis.

In the domain *Work organization and job content*, we found that higher levels of *influence at work*, *influence on working hours*, *possibilities for development*, *role clarity*, *predictability*, and *possibilities for performing work tasks* on the one side were associated with a reduced risk of LTSA on the other side. For these measures, hazard ratios varied between 0.71 and 0.92 for a 1 SD increase in the predictors. CIs for the measures *possibilities for development* and *role clarity* did not depart from unity. Fig. 2 also shows that higher levels of *role conflicts* and *unnecessary work tasks* were associated with an increased risk of LTSA during follow-up. All findings in this domain were in accordance with our hypotheses.

Within the domain *Interpersonal relations: cooperation and leadership* all measures were associated with a reduced risk of LTSA at follow-up. These findings are in compliance with our hypotheses. Hazard ratios for a 1 SD increase in the predictors varied between 0.73 and 0.94 and for five of the associations, the CI departed from unity, while the CI did not depart from unity for the other five associations.

Finally, we found that all measures within the domain *Reactions to the work situation* were associated with risk of LTSA during follow-up and the direction of the observed associations were in accordance with our hypotheses. Fig. 2 shows that higher levels of *job insecurity*, *stress*, and *conflict between work and private life* predicted increased risk of LTSA and these hazard ratios varied between 1.40 and 1.49. For the other measures in this domain, we found that higher scale values at baseline were associated with lower risk of LTSA during follow-up. For these latter associations, the hazard ratios for a 1 SD increase in the predictors varied between 0.74 and 0.81. For all of the observed associations in this domain the CIs departed from unity.

Fig. 2 shows that 22 of the 32 associations we statistically significant. Of the 22 statistically significant associations, 20 remained statistically significant after Benjamini–Hochberg correction for multiple testing.

## Discussion

In this study, we investigated the predictive validity of 32 multi-item scales and single items from the DPQ against two criteria variables, onset of depressive disorders and onset of LTSA. We deployed three conditions in our assessment of predictive validity: (i) did the associations point in the directions, hypothesized in Table 1? and (ii) were the associations practically relevant? and (iii) were the associations statistically significant?

The analyses using *onset of depressive disorders* at follow-up as criterion provided support for the predictive validity of most measures of the DPQ. The direction of all associations complied with the hypotheses presented in Table 1. With two exceptions—*cognitive demands* and *work without boundaries—*we found that the risk estimates for the association between the 32 DPQ-measures and onset of depressive disorders deviated clearly from unity and, accordingly, we also observed statistically significant associations in 30 of the 32 associations, which further supports the predictive validity of the measures of the DPQ in the present study population.

The analyses using *onset of LTSA* during follow-up as criterion also provided support for the predictive validity of the measures of the DPQ. For 29 of the 32 measures of the DPQ that we analyze in this study, the association with LTSA pointed in the direction hypothesized in Table 1. For the multi-item scales *work without boundaries* and *quantitative demands*, we found that the direction of the observed associations did not comply with our hypotheses. From a previous study (Clausen *et al.*, 2014a), we expected that higher *quantitative demands* would predict lower risk of LTSA, but the results indicated, that higher *quantitative demands* predicted increased risk of LTSA. However, this finding is in accordance with the propositions of the Job Demands-Resources model that posits that higher levels of job demands will associate with negative health-related outcomes (Schaufeli and Bakker, 2004). Moreover, we found that one measure—*cognitive demands—*did not depart sufficiently from the reference category to provide an indication of the direction of the observed association. Finally, we observed statistically significant associations in 20 of the 32 associations, which further supports the predictive validity of these measures of the DPQ in the present study population.

When interpreting the results from the analyses of the predictive validity of the measures of the DPQ, it is also important to assess the practical relevance of the observed associations. Overall, the results indicate that the DPQ-measures are stronger predictors of onset of depressive disorders than onset of LTSA. For both outcomes, however, we found that an increase of 1 SD in the DPQ-measures was associated with clear increases or reductions in the risk of the two outcomes. A closer inspection of the association between DPQ-measures and risk of onset of depressive disorders show that 11 of the 32 DPQ-measures predict and increased risk of depressive disorders during follow-up. Of these 11 associations, two exhibited odds ratios that were smaller than 1.3, one had an odds ratio between 1.3 and 1.5 and the remaining eight associations exhibited odds ratios between 1.5 and 2.44. For the 21 DPQ-measures that predicted a reduced risk of depressive disorders during follow-up, two exhibited odds ratios between 0.7 and 1.0, and the remaining 17 associations had odds ratios below 0.7 and 0.5. An inspection of the associations between the DPQ-measures and risk of onset of LTSA show that nine of the DPQ-measures predicted an increased risk of LTSA. Four measures exhibited hazard ratios between 1.1 and 1.3 and for five DPQ-measures we found hazard ratios between 1.3 and 1.5. For the 23 DPQ-measures that predicted a reduced risk of LTSA during follow-up, three associations exhibited hazard ratios between 1 and 0.9 and the hazard ratios for the remaining 20 associations varied between 0.9 and 0.7. Accordingly, this inspection of the magnitudes of the observed risk estimates in the associations between the DPQ-measures and the two outcomes support the predictive validity of the DPQ-measures as an increase of 1 SD for most of the DPQ-measures is predictive of onset of depressive disorders and onset of LTSA. With few exceptions, the observed risk estimates exceed a factor 0.1, which implies that the results must be considered practically relevant as a reduction in risk of depressive disorders or LTSA of 0.1 or more for an increase/decrease in the relevant DPQ-measures may contribute significantly to enhancing the health and well-being of workers at the level of individuals, workplaces, and societies.

In the present study, we found that adverse psychosocial working conditions were associated with onset of depressive disorders and onset of LTSA. It was beyond the scope of the study to investigate potential interrelations between the measures of the DPQ and their associations to the two outcome variables. It could for instance be expected that leadership resources would be associated with measures related to cooperation between colleagues and measures within the domains *Demands at work*, and *Work organization and job content* (Berthelsen *et al.*, 2018). It could also be expected that DPQ-measures within the domains *Demands at work*, *Work organization and job content*, and *Interpersonal relations* might be associated with the DPQ-measures in the domain *Reactions to the work* situation (Schaufeli and Bakker, 2004; Clausen and Borg, 2011). Moreover, it is plausible that the two outcome variables are interrelated, for example that depressive disorders may constitute a potential mediator in the association between DPQ-measures and LTSA (Thorsen *et al.*, 2013). Against this background, it must be noted, however, that we designed this study to test the predictive validity of the individual DPQ-measures and not for investigating potential mechanisms or drawing causal inference on the prospective association between psychosocial working conditions and the two outcomes.

The results reported in the present study complement previous findings from the analysis of the validity and the reliability of the DPQ (Clausen *et al.*, 2019). Thus, we have now analyzed the test–retest reliability, the internal consistency reliability, and the construct validity (with particular focus on the factorial validity, the discriminant validity, and the predictive validity) of the measures of the DPQ. The results from these two studies generally support that the DPQ offers a valid and reliable instrument for the measurement of psychosocial working conditions. That said, the results from the previous study (Clausen *et al.*, 2019) showed, that although the factorial validity was satisfactory *within* each of the 14 job groups that were included in the study, not all multi-item scales were factorially invariant, which implies that the factor loadings of individual items may vary across job groups.

### Strengths and limitations

It is a strength of the study that we use two conceptually different outcomes in the analyses and that one is measured using self-reports and the other is measured in a national register. The self-reported outcome of depressive disorders allows us to test the predictive validity using an affective outcome as criterion. This analysis provided support for most of the DPQ-measures. It cannot be ruled out, however, that the magnitude of these associations may be inflated due to common methods biases (Podsakoff *et al.*, 2003), although we have reduced the risk of common methods bias in the analysis to some extent through the prospective design of the study. Against this background, it is a strength of the study that we have used a register-based measure of sickness absence as this eliminates any risk of common methods biases (Podsakoff *et al.*, 2003).

In the analyses of depressive disorders, we used a follow-up period of 6 months. It can be discussed whether this is the correct duration of follow-up. An advantage of this duration of follow-up is that it reduces the risk of misclassification (Rothman and Greenland, 1998) as the working conditions are less likely to change over shorter rather than longer follow-up periods and in spite of the relatively short follow-up, the results showed clear associations the DPQ-measures and the applied measure of depressive disorders.

It may be considered a limitation of the study that we did not analyze all 32 DPQ-measures together in one model. However, since the DPQ-measures are correlated within each of the four overall domains in the DPQ, we have refrained from undertaking such an analysis due to the risk of multicollinearity.

Finally, it can be argued that our analysis of the predictive validity of the DPQ-measures is over-adjusted since we excluded participants with depressive symptoms at baseline and participants with prior LTSA. We decided, however, to conduct the tests as proper prospective analyses, which implies that we must take the baseline levels of the outcomes into account. Moreover, by excluding respondents with depressive disorders at baseline and respondents with prior LTSA, we also take indicators of baseline health into account in the respective analyses.

## Conclusions

We investigated the predictive validity of the DPQ using two criteria variables: onset of depressive disorders and onset of LTSA. The analyses provided support for the predictive validity of most DPQ-measures, and showed that, overall, the associations were stronger when analyzing onset of depressive disorders than onset of LTSA. Adding to the previously reported results on the construct validity and the reliability of the DPQ-measures (Clausen *et al.*, 2019), the results from the present article suggest that the DPQ constitutes a useful tool for identifying risk factors in the psychosocial work environment. We acknowledge that the test of the predictive validity of questionnaire-based measures is an on-going process and we recommend that the predictive validity of the DPQ-measures is continuously monitored in future studies using the DPQ.

## Supplementary Material

wxac069_suppl_Supplementary_Material

## Data Availability

The data underlying this study will be shared on reasonable request to the corresponding author.
