# Supplementary material for: The Predictive Validity of the Danish Psychosocial Work Environment Questionnaire With Regard to Onset of Depressive Disorders and Long-Term Sickness Absence
Source: Ann Work Expo Health. 2022 Oct 15;67(2):195–207. doi: 10.1093/annweh/wxac069 (PMC9923041; doi:10.1093/annweh/wxac069)
Supplement: wxac069_suppl_Supplementary_Material [file wxac069_suppl_supplementary_material.docx]

**ONLINE SUPPLEMENTARY MATERIAL**

**The predictive validity of the Danish Psychosocial Work Environment Questionnaire with regard onset of depressive disorders and long-term sickness absence**

Thomas Clausen, PhD^1^, Karl Bang Christensen, PhD ^2^, Jeppe Karl Sørensen, MSc^1^, Jakob B Bjorner, PhD ^1,2,3^, Ida EH Madsen, PhD ^1^, Vilhelm Borg, MSc^1^ & Reiner Rugulies, PhD ^1,2^

^1^ National Research Centre for the Working Environment, Copenhagen, Denmark

^2^ Department of Public Health, University of Copenhagen, Denmark

^3^ Optum Patient Insights, Johnston, RI, USA

# Contents

[Appendix 1: Overview of dimensions and items in the Danish Psychosocial Work Environment Questionnaire 2](#_Toc107408459)

# Appendix 1: Overview of dimensions and items in the Danish Psychosocial Work Environment Questionnaire (DPQ)

| **Dimension** | **Items** | **Cronbach’s α** | |
| --- | --- | --- | --- |
| **Domain: Demands at work** | | |  |
| **Quantitative demands** | 1. How often is it the case that you do not have time to complete all your work tasks? ^a^ 2. How often do you receive unscheduled work tasks that place you under time pressure? ^a^ 3. How often do you have deadlines that are hard to meet? ^a^ 4. Do you get behind with your work? ^a^ | 0.84 | |
| **Work pace** | 1. Is the pace of work so fast that it affects the quality of your work? ^a^ 2. Do you have to work very fast? ^a^ | - | |
| **Emotional demands** | 1. Are you placed in emotionally demanding situations at work? ^a^ 2. As a result of your work, do you come into contact with people who oppose you or are aggressive towards you? ^a^ 3. Do you have to deal with relationships at work that are emotionally challenging? ^a^ 4. As a result of your work, do you have contact with people who are in difficult situations (e.g. people affected by a serious illness, accidents, grief, crises or social problems)? ^a^ | 0.83 | |
| **Demands to conceal feelings** | 1. Do you have to be friendly and receptive towards everyone, regardless of how they treat you? ^a^ 2. Does your job require that you do not display your feelings? ^a^ | - | |
| **Cognitive demands** | 1. Does your work require your undivided attention? ^b^ 2. Do you have to process large amounts of information in your work? ^b^ 3. Does your job require you to make complicated decisions? ^b^ 4. Do you have to pay attention to many things at once in your job? ^a^ | 0.80 | |
| **Work without boundaries** | 1. How often are you 'on-call' to be available for work outside of your normal working hours? ^a^ 2. How often do you work at home outside of your normal working hours, e.g. in the evening, during weekends or during holidays? ^a^ 3. How often do you work on days when you are off from work, e.g. on weekends, holidays or vacations? ^a^ 4. How often does your job require you to work overtime, i.e. beyond your agreed or expected working hours? ^a^ | 0.82 | |
| **Dimension** | **Items** | **Cronbach’s α** | |
| **Domain: Work organization and job content** | | |  |
| **Influence at work** | 1. Do you have any influence on how you carry out your work tasks? ^b^ 2. Do you have sufficient authority to deal with the responsibilities you have in your work? ^b^ 3. Is it possible for you to make important decisions about your work? ^b^ 4. Do you have any influence on the order in which you carry out your work tasks? ^b^ | 0.87 | |
| **Influence on working hours** | 1. Do you have influence on your working hours, e.g. when you arrive at work or when you go home from work? ^b^ 2. Do you have any influence on when you take breaks during the course of the working day? ^b^ 3. Do you have any influence on when you take your vacation? ^b^ | 0.81 | |
| **Possibilities for development** | 1. Does your work provide you with opportunities for developing your skills? ^b^ 2. Do your work tasks vary a lot? ^b^ 3. Do you have possibilities to learn something new through your work? ^b^ 4. Do you have good opportunities for further training and education? ^b^ | 0.84 | |
| **Role clarity** | 1. Are there clear goals for your work tasks? ^b^ 2. Do you know exactly what is expected of you at work? ^b^ 3. Do you know when you have carried out your job well? ^b^ 4. Do you know exactly what your responsibilities are? ^b^ | 0.81 | |
| **Role conflicts** | 1. Do you have to do things in your work that you feel should be done differently? ^b^ 2. Are there any conflicting demands in your work? ^b^ 3. Does your job involve tasks that conflict with your personal values? ^b^ 4. Do you sometimes have to end a task even though you do not feel you have completed it? ^b^ | 0.78 | |
| **Predictability** | 1. Do you receive timely information about e.g. important decisions, changes and plans for the future at your place of work? ^b^ 2. Are you informed well in advance if changes are made to your work tasks? ^b^ 3. Are you informed well in advance of changes to whom you will be working with? ^b^ 4. Are you informed well in advance if there are changes to your working hours? ^b^ | 0.79 | |

| **Dimension** | | **Items** | **Cronbach’s α** | |
| --- | --- | --- | --- | --- |
| **Possibilities for performing work tasks** | | 1. Do your working conditions allow you to carry out your work satisfactorily? ^b^ 2. Do you have the tools you need (e.g. technical assistive devices, tools, machinery, IT solutions, etc.) for you to do your job satisfactorily? ^b^ 3. Are there enough employees at work for you to do your job satisfactorily?^b^ 4. Can you perform your work tasks to a level of quality that you are satisfied with? ^b^ | 0.81 | |
| **Unnecessary work tasks** | | 1. Do you spend time on work tasks that you have difficulty seeing the purpose with? ^b^ 2. Are you placed in situations at work that are unnecessarily difficult to deal with? ^b^ 3. Is your work made more difficult than necessary due to poor work procedures? (b) 4. Do you have to do work tasks that you think are unnecessary? ^b^ | 0.81 | |
| **Domain: Interpersonal relations: cooperation and leadership** | | | |  |
| **Social support from colleagues** | 1. Can you get practical help with your work from colleagues if you need it? ^b^ 2. Can you get advice and guidance from your colleagues if you need it? ^b^ 3. Can you talk to your colleagues about it if you experience difficulties at work? ^b^ 4. Are you and your colleagues attentive to each other's wellbeing? ^b^ | | 0.82 | |
| **Cooperation between colleagues within teams, departments, or groups** | 1. Do you and your colleagues help each other if someone has too much to do? ^b^ 2. Is there a sense of community and cohesion between you and your colleagues? ^b^ 3. Do you and your colleagues work well together when problems emerge which require cooperation among you? ^b^ 4. Do you and your colleagues agree on what is most important in your work tasks? ^b^ | | 0.82 | |
| **Trust between colleagues** | 1. Do you trust the ability of your colleagues to do their job well? ^b^ 2. Can you express your views and feelings to your closest colleagues? ^b^ 3. In general, do you and your colleagues trust one another? ^b^ 4. Do you and your colleagues keep each other informed about things that are important for you to do your job well? ^b^ | | 0.81 | |
| **Social support from management** | 1. Can you get practical help with your work from your immediate supervisor if you need it? ^b^ 2. Can you talk with your immediate supervisor about difficulties you experience at work? ^b^ 3. Does your immediate supervisor follow up on conversations about any difficulties you have experienced at work? ^b^ 4. Can you get advice and guidance from your immediate supervisor if you need it? ^b^ | | 0.90 | |
| **Dimension** | | **Items** | **Cronbach’s α** | |
| **Quality of leadership** | 1. Does your immediate supervisor give high priority to the wellbeing of employees in the workplace? ^b^ 2. Is your immediate supervisor good at communicating clear goals for the work of you and your colleagues? ^b^ 3. Is your immediate supervisor good at resolving conflicts? ^b^ 4. Is your immediate supervisor good at motivating the employees? ^b^ | | 0.91 | |
| **Cooperation with immediate supervisor** | 1. Is the relationship between your immediate supervisor and you and your co-workers characterized by mutual respect and recognition? ^b^ 2. Does your immediate supervisor have a clear understanding of the work tasks that you and your co-workers perform? ^b^ 3. Does your immediate supervisor take the needs and views of you and your co-workers into consideration when making decisions? ^b^ 4. Does your immediate supervisor contribute to solving everyday problems? ^b^ | | 0.88 | |
| **Justice in the workplace** | 1. Are conflicts resolved in a fair way? ^b^ 2. Can one get a clear reason when important decisions are made in your workplace? ^b^ 3. Does the management at your workplace respect you? ^b^ 4. Does the management at your workplace treat you fairly? ^b^ | | 0.85 | |
| **Involvement of employees** | 1. Does the management encourage you and your colleagues to come up with ideas for improvements? ^b^ 2. Do employees and managers work well together to improve work procedures? ^b^ 3. Are suggestions for improvements treated seriously by the management in the workplace? ^b^ | | 0.90 | |
| **Changes in the workplace** | 1. Have any major changes been implemented at your workplace during the last two years (e.g. a restructuring of the workplace or layoffs)? ^c^   If “Yes”, the following questions should be asked:   1. Did the management inform the employees sufficiently about the changes in the workplace? ^b^ 2. Have the employees been sufficiently involved in relation to the changes? ^b^ 3. Are you generally satisfied with the way the management dealt with the changes? ^b^ 4. Do you understand the management's reasons for implementing the changes? ^b^ | | 0.86 | |
| **Recognition** | 1. Are your efforts recognized and appreciated at your place of work? ^b^ | | - | |
| **Dimension** | | **Items** | **Cronbach’s α** | |
| **Domain: Reactions to the work situation** | | | |  |
| **Experience of meaning at work** | 1. Do you feel motivated and engaged in your work? ^b^ 2. Are your work tasks meaningful? ^b^ 3. Do you think that your work tasks are interesting and inspiring? ^b^ 4. Does your work give you self-confidence and job satisfaction? ^b^ | | 0.90 |  |
| **Commitment to the workplace** | 1. Would you recommend others to apply for a job at your workplace? ^b^ 2. Do you tell your friends that your workplace is a good place to work? ^b^ 3. Are you proud of working at your workplace? ^b^ 4. Does your workplace inspire you to do your best? ^b^ | | 0.92 |  |
| **Work engagement** | 1. At my work, I feel bursting with energy ^h^ 2. I am enthusiastic about my job ^h^ 3. I feel happy when I am working intensely ^h^ 4. At my job, I feel strong and vigorous ^h^ 5. I am proud of the work that I do ^h^ 6. I am immersed in my work ^h^ 7. When I get up in the morning, I feel like going to work ^h^ 8. My job inspires me ^h^ 9. I get carried away when I am working ^h^ | | 0.95 |  |
| **Job insecurity** | 1. Do you worry about becoming unemployed? ^b^ 2. Do you worry that it could be difficult to find another job if you become unemployed? ^b^ 3. Do you worry about being transferred to another job against your will? ^b^ | | 0.79 |  |
| **Self-reported stress** | 1. How often have you felt stressed within that last two weeks? ^i^   If “All the time”, “Often”, “Sometimes”, or “Rarely”, the following questions should be asked:   1. What was the most significant source of your stress? ^j^ | | - |  |
| **Job satisfaction** | 1. Overall, how satisfied are you with your job? ^k^ | | - |  |
| **Overall assessment of the psychosocial work environment** | 1. Overall, how satisfied are you with the social and organizational work environment in your workplace? ^l^ | | - |  |
| **Dimension** | | **Items** | **Cronbach’s α** | |
| **Conflict between work-life and private life** | 1. Does your job demand so much of your energy that it has a negative effect on your private life? ^b^ 2. Does your job demand so much of your time that it has a negative effect on your private life? ^b^ 3. Does your job demand so much of your attention that it has a negative effect on your private life? ^b^ | | 0.89 |  |

a: Response options: “Always”; “Often”; “Sometimes”; “Rarely”; “Never/almost never”

b: Response options: “To a very large extent”; “To a large extent”; “Somewhat”; “To a small extent”; “To a very small extent”

c: Response options: “Yes, several times”; “Yes, one time”; “No”

d: Response options: “Yes, daily or almost daily”; “Yes, weekly”; “Yes, monthly”; “Yes, occasionally”; “No”

e: Response options: “Overall management/Business owner”; “Immediate supervisor”; “Colleagues”; “Subordinates”; “Customers, clients, patients, pupils, relatives (with ‘relatives’ we think of relatives to pupils, clients or patients)”; “The threat has/threats have been put forward anonymously”

f: Response options: “Overall management/Business owner”; “Immediate supervisor”; “Colleagues”; “Subordinates”; “Customers, clients, patients, pupils, relatives (with ‘relatives’ we think of relatives to pupils, clients or patients)”

g: Response options: “At my workplace”; “Outside of my workplace, e.g. at home or in town”; “Via social media”; “By telephone, SMS, email or letter”; “Other”

h: Response options: “Never”; “Almost never”; “Rarely”; “Sometimes”; “Often”; “Very often”; “Always”

i: Response options: “All the time”; “Often”; “Sometimes”; “Rarely”; “Never”

j: Response options: “Work”; “Private life”; “Both work and private life”

k: Response options: A scale from 0 to 10, where 0 denotes the lowest possible level of job satisfaction and 10 denotes the highest possible level of job satisfaction.

l: Response options: A scale from 0 to 10, where 0 denotes the lowest possible assessment of the psychosocial work environment and 10 denotes the highest possible assessment of the psychosocial work environment.

**Coding of items and multi-item scales**

With the exception of the six dimensions measured within the domain *Conflicts in the workplace*, all dimensions (scales and single items) were measured by scales ranging from 0 to 100. Scale scores were calculated by recoding item scores from 0 to 100 and averaging the scores for items within each scale. For each scale, the score of 100 indicates the highest level of the measured dimension.

Response options for items with five-point Likert scales were scored as follows:

| Response option | Score |
| --- | --- |
| To a very large extent // Always | 100 |
| To a large extent // Often | 75 |
| Somewhat // Sometimes | 50 |
| To a small extent // Rarely | 25 |
| To a very small extent // Never/almost never | 0 |

Response options for items in the scale ‘Work engagement’ were scored as follows:

| Response option | Score |
| --- | --- |
| Never | 0.0 |
| Almost never | 16.7 |
| Rarely | 33.3 |
| Sometimes | 50.0 |
| Often | 66.7 |
| Very often | 83.3 |
| Always | 100.0 |

Response options for the two dimensions *Job satisfaction* and *Overall assessment of the psychosocial work environment* were scored on a scale from 0 to 10. To align scores on these two dimensions with a scale ranging from 0 to 100, responses on the original response scale were multiplied with 10.

In multi-item scales we added the score for the chosen response option for each item and divided the sum score with the number of items in the multi-item scale. In cases where respondents had only responded to some of the items making up a given scale, scales values were calculated if the respondent had responded to half of the items or more.
